# Preference for and learning of amino acids in larval *Drosophila*

**DOI:** 10.1242/bio.020412

**Published:** 2017-02-13

**Authors:** Nana Kudow, Daisuke Miura, Michael Schleyer, Naoko Toshima, Bertram Gerber, Teiichi Tanimura

**Affiliations:** 1 Department of Biology, Faculty of Science, Kyushu University, Motooka 744, Fukuoka 819-0395, Japan; 2 Division of Biological Science, Graduate School of Systems Life Sciences, Kyushu University, Motooka 744, Fukuoka 819-0395, Japan; 3 Leibniz Institute for Neurobiology (LIN), Department Genetics of Learning and Memory, Brenneckestrasse 6, Magdeburg 39118, Germany; 4 Center for Behavioral Brain Sciences (CBBS), Universitätsplatz 2, 39106 Magdeburg, Germany; 5 Otto von Guericke University Magdeburg, Institute for Biology, Universitätsplatz 2, Magdeburg 39106, Germany

**Keywords:** *Drosophila*, Amino acid, Gustation, Preference, Learning

## Abstract

Relative to other nutrients, less is known about how animals sense amino acids and how behaviour is organized accordingly. This is a significant gap in our knowledge because amino acids are required for protein synthesis − and hence for life as we know it. Choosing *Drosophila* larvae as a case study, we provide the first systematic analysis of both the preference behaviour for, and the learning of, all 20 canonical amino acids in *Drosophila*. We report that preference for individual amino acids differs according to the kind of amino acid, both in first-instar and in third-instar larvae. Our data suggest that this preference profile changes across larval instars, and that starvation during the third instar also alters this profile. Only aspartic acid turns out to be robustly attractive across all our experiments. The essentiality of amino acids does not appear to be a determinant of preference. Interestingly, although amino acids thus differ in their innate attractiveness, we find that all amino acids are equally rewarding. Similar discrepancies between innate attractiveness and reinforcing effect have previously been reported for other tastants, including sugars, bitter substances and salt. The present analyses will facilitate the ongoing search for the receptors, sensory neurons, and internal, homeostatic amino acid sensors in *Drosophila*.

## Abstract (precis)

**Summary:** *Drosophila* larvae show positive preference but for a few individual amino acids. The preference partially changes depending on larval stage and deprivation. In learning experiments, all 20 tested amino acids were equally rewarding.

## INTRODUCTION

Amino acids are required for protein synthesis and are therefore essential for all organisms. Animals either need to break down ingested protein to obtain amino acids, or synthesize them themselves. Thus, the internal monitoring of amino acid demand and the organization of behaviour to secure their supply is important to any animal, and certainly to man as well. Relative to other nutrients, however, less is known about how amino acids are sensed and how the search for and the behaviour towards amino acids are organized. Choosing larval *Drosophila* as a case study, we provide the first systematic analysis of both the preference behaviour for, and the learning of, all 20 canonical amino acids, including those classified as essential for egg-production in adult *Drosophila* (Sang and King, 1961).

Larvae are the feeding and growth stages of holometabolous insects, and as such lend themselves to studies of chemosensory behaviour (Gerber and Stocker, 2007; Gerber et al., 2009; Apostolopoulou et al., 2015). *Drosophila* third-instar larvae show preference for various sugars and low salt concentrations, and avoid ‘bitter’ compounds such as quinine and high salt concentrations. These tastants are furthermore effective as rewards and punishments, respectively, in odour-taste associative learning paradigms (Niewalda et al., 2008; Schipanski et al., 2008; El-Keredy et al., 2012; Apostolopoulou et al., 2014a,b; Kim et al., 2015). Regarding amino acid processing in third-instar *Drosophila* larvae, it is only known that they preferentially ingest amino acid-rich soybean rather than other tested foods (Ryuda et al., 2008), and that aspartic acid is a strong reward (Schleyer et al., 2015) (on glycine as a reward in honey bees, see Kim and Smith, 2000). Furthermore, Croset et al. (2016) recently reported that amino acids differ in the level of preference/avoidance they induce, and that high concentrations of some amino acids can reduce feeding when added to sucrose. The authors showed that half of the 19 individually tested L-amino amino acids, but none of the 10 tested D-amino acids, physiologically activate two gustatory sensory neurons of the larva that express the Ir60c chemoreceptor. Activating these neurons was sufficient to inhibit sucrose feeding, while blocking synaptic output from them lifted the inhibition of sucrose feeding by high concentrations of alanine or of arginine (but not lysine). Such block of synaptic output also levelled out the preference for sucrose alone over a mixture of sucrose and high-concentration alanine. Despite these advances, a comprehensive picture of how amino acids are processed and how the various types of behaviour related to amino acids are organized is still lacking for third-instar larvae, and indeed nothing is known about these processes in earlier larval instars. In this study we decided to ask for all 20 canonical amino acids: (i) which of them support preference behaviour in first- and/or third-instar larvae; (ii) whether amino acid preference in third-instar larvae changes upon starvation; and (iii) which amino acids in third-instar larvae are effective as a reward.

## RESULTS

### Preference differs between amino acids and depends on stage and starvation

**Fig. 1.**
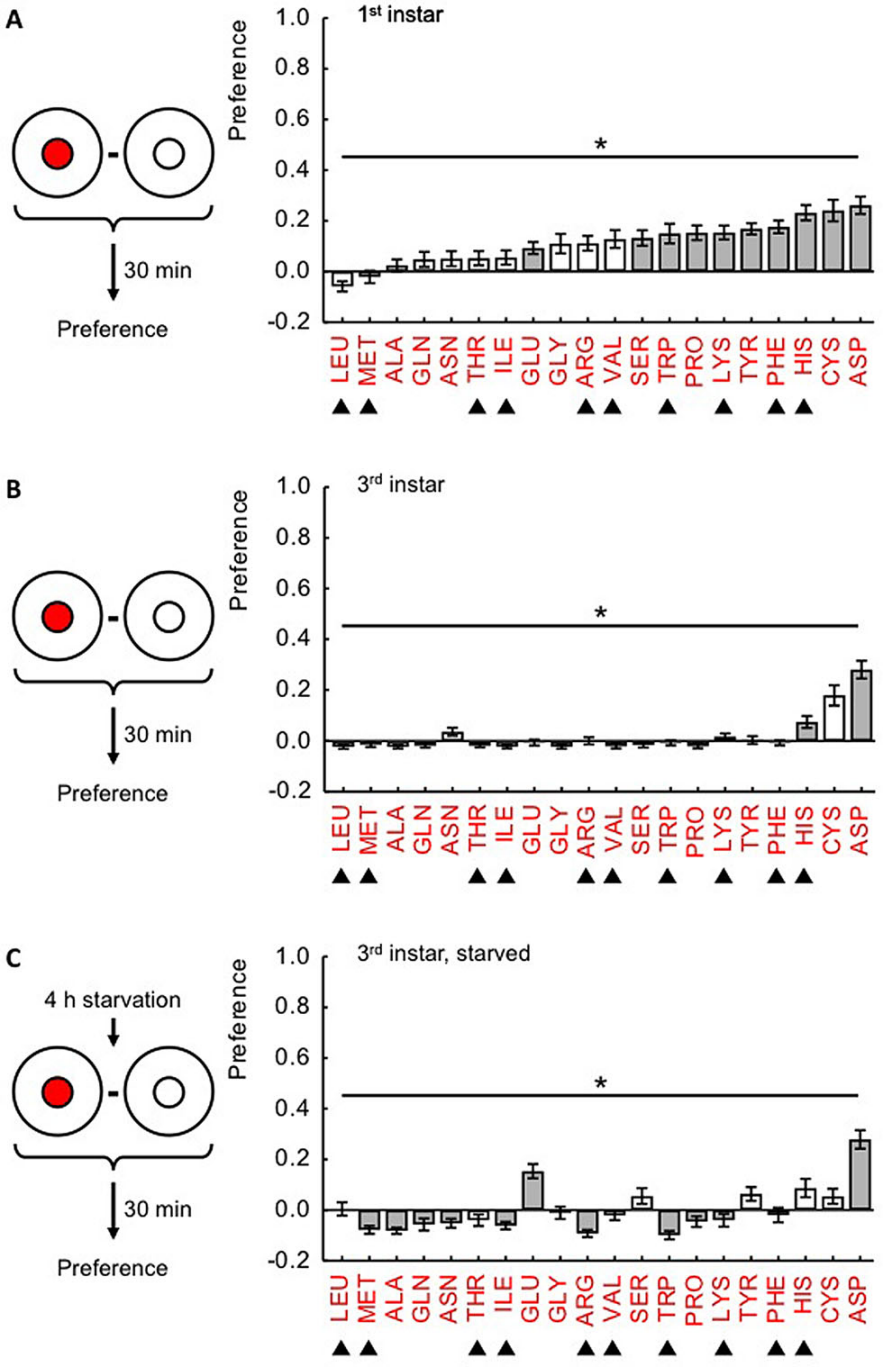
**Innate preference differs across 20 individual amino acids and depends on stage and starvation.** Groups of larvae are placed on a Petri dish that contains in its centre either one of 20 amino acids (small red circle) or plain agar (small white circle), in both cases surrounded by plain agar. After 30 min, the larvae are counted and preferences are calculated. For the underlying ratios of animals in the inner circle versus total, see Fig. S2. (A) Preferences of first-instar larvae differ across amino acids (*P*<0.05, H=127). From the 20 amino acids tested, GLU, SER, TRP, PRO, LYS, TYR, PHE, HIS, CYS and ASP are significantly preferred as indicated by shading of the bars (*P*<0.05 in one-sample sign tests, corrected for multiple comparisons according to Bonferroni-Holm). (B) Preferences of third-instar larvae also differ across amino acids (*P*<0.05, H=167.5). Only HIS and ASP are significantly preferred. LEU, GLY, VAL, SER and PRO are weakly yet significantly avoided. Details as in A. (C) After 4 h of food-deprivation in distilled water, preference scores of third-instar larvae differ across amino acids (*P*<0.05, H=166.5), with GLU and ASP being significantly preferred. MET, ALA, GLN, ASN, ILE, ARG, TRP, PRO and LYS are significantly avoided at weak to moderate levels. Details as in A. For amino acid abbreviations, see Materials and Methods. Filled triangles indicate amino acids classified as essential by Sang and King (1961). Bars and error bars display mean±s.e.m. Sample sizes for each case respectively are: (A) 33-35, (B) 35, (C) 35.

Given that proteins are important during larval growth, we started out by testing the innate preference towards 20 individual amino acids in first-instar *Drosophila* larvae, i.e. at the stage during which growth is particularly rapid. The larvae were tested just after hatching and had no previous access to food. The preference scores differ across amino acids (Fig. 1A; for the underlying ratios of the number of animals in the inner circle versus total, see Fig. S2A). At the concentration used, we observed significant preference for half of the tested amino acids, including histidine (HIS), cysteine (CYS), and aspartic acid (ASP). We did not observe any significant avoidance towards any amino acid at the used concentration.

Larval growth largely ceases during the third-instar stage. Fittingly, we found that the preference scores for amino acids were often lower than in first-instar larvae and indeed were significantly positive only for HIS and ASP at the given concentration (Fig. 1B; for the underlying ratios of animals in the inner circle versus total, see Fig. S2B); preference for CYS just failed to reach significance. For some of the amino acids we observed rather weak, yet statistically significant, avoidance.

We next asked whether, in third-instar larvae, starving the animals before the test would alter preference scores. Larvae continued to show preference for ASP under starved conditions (Fig. 1C; for the underlying ratios of animals in the inner circle versus total, see Fig. S2C). Different from the non-starved condition, preference scores were found to be significantly positive for the used concentration of glutamic acid (GLU) in starved larvae. For nine amino acids, unexpectedly, we observed weak to moderate aversion. Thus, there did not appear to be a general upshift in preferences caused by starvation; rather, the data suggest that starvation alters the profile of liked and disliked amino acids.

Across the three above-described experiments, the preference scores were not consistently different between essential and non-essential amino acids when tested individually (Fig. 1A-C). In first-instar larvae, however, we found preference scores for a mixture of all 10 essential amino acids to be stronger than for a mixture of the remaining 10 non-essential amino acids (Fig. S3). We also found no consistent effect of chemical properties such as polarity or acidity on larval behaviour (see also Croset et al., 2016).

### Reward strength does not differ between amino acids

**Fig. 2.**
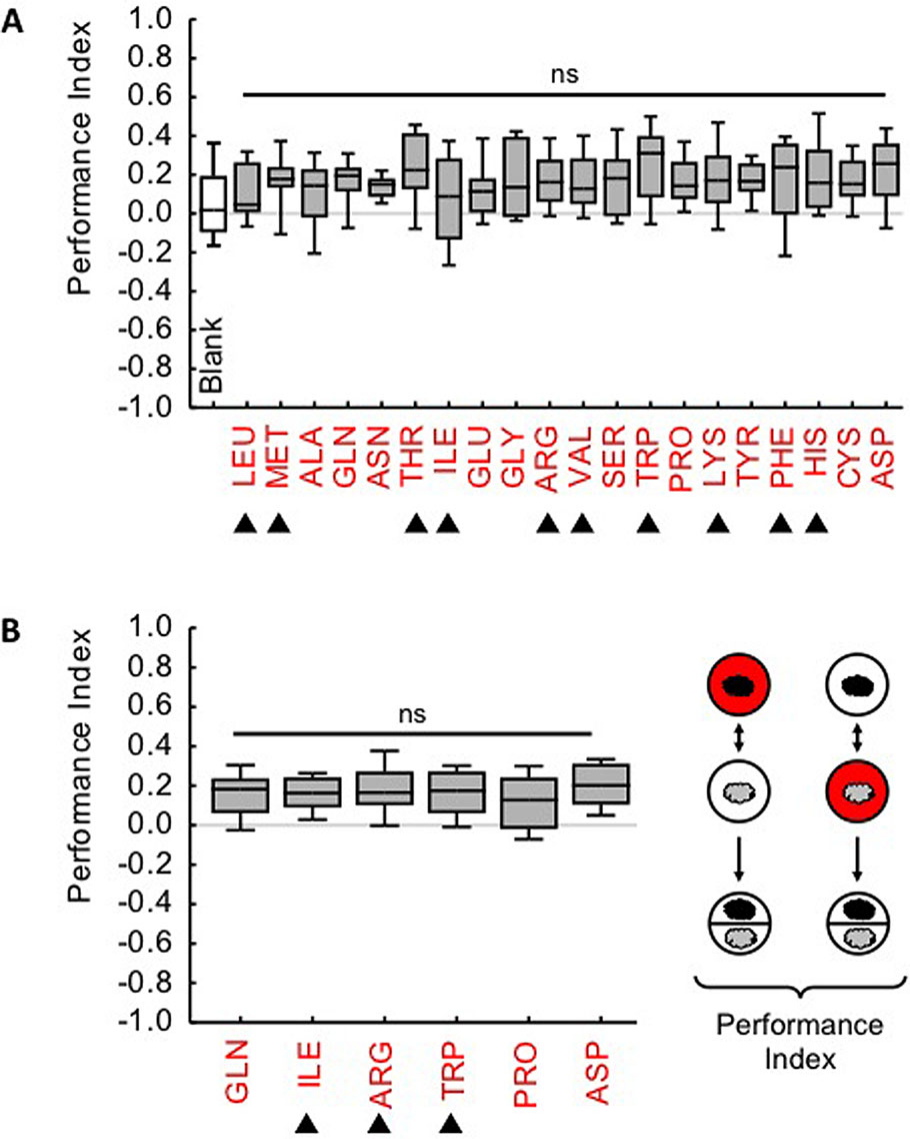
**All 20 individual amino acids are equally rewarding.** The sketch at the bottom-right depicts the learning paradigm. One odour (indicated by a cloud) was presented together with a candidate amino acid reward in the Petri dish (red fill of circle), while another odour was presented alone (white fill). For an effective reward, the larvae show a higher preference for the respectively rewarded odour (Fig. S4). This difference in preference indicates associative memory and is quantified by the Performance Index displayed in (A) and (B). (A) For all 20 amino acids the larvae show positive, i.e. appetitive memory scores; these scores are statistically indistinguishable between amino acids (*P*>0.05, H=15.3). The pooled memory scores from all amino acids are significantly different from zero (*P*<0.05 in one-sample sign tests, corrected for multiple comparisons according to Bonferroni-Holm) and are different from the negative control tested with tasteless agar only (‘Blank’) (Fig. S5). (B) To confirm the lack of difference between amino acids, we selected six amino acids including those yielding the relatively lowest and highest scores in (A) and repeated the experiment. We confirm that all the used amino acids are equally rewarding (*P*>0.05, H=3.9). Box plots show the median as the middle line, and 25 and 75%, and 10 and 90% quantiles as box boundaries and whiskers, respectively. Shaded boxes indicate that the pooled values of all amino acids are significantly different from zero. Filled triangles indicate amino acids classified as essential by Sang and King (1961). Sample sizes for each case respectively are: (A) 16-20, (B) 20.

Given the differences in preference observed for different amino acids, we wondered whether amino acids would likewise differ in their strength as a reward. We trained third-instar larvae such that one odour was paired with either one of the 20 amino acids, while a second odour was presented alone (given the duration of the training procedure and the exposure to only one amino acid during training the animals may be regarded as partially and mildly amino acid-deprived at the moment of testing). After such training, we tested the animals' choice between the two odours. In all cases choice was biased in favour of the respective amino acid-paired odour, as quantified by the Performance Index (Fig. 2A; for the underlying preference scores see Fig. S4A, for a display of the pooled data of all amino acids, see Fig. S5). Notably, memory scores were statistically indistinguishable between amino acids, a finding that was confirmed in an independent repetition of a subset of the used amino acids (Fig. 2B; for the underlying preference scores see Fig. S4B). We therefore suggest that individual amino acids have indistinguishable reward strength. We further note that memory scores did not systematically differ between essential and non-essential amino acids.

## DISCUSSION

The present study systematically analyses both the preference behaviour for, and the rewarding effects of, individual amino acids in larval *Drosophila*.

We found that taste preferences for amino acids differ according to the kind of amino acid, both in first-instar and in third-instar larvae (Fig. 1). In the mosquito *Aedes aegypti* differences in preference across amino acids have also been reported (Ignell et al., 2010). Notably, we did not find any consistent relationship between the physico-chemical properties of the amino acids (e.g. polarity or acidity) with preference behaviour (see also Croset et al. 2016). Therefore, it seems to be unlikely that those properties are major determinants of larval preference.

Our data suggest that the profile of amino acid preference changes across larval instars and that starvation during the third instar also alters this profile (Fig. 1). The fact that these modulations in amino acid preference do not affect all amino acids in the same way implies some specificity in how amino acids are sensed and/or processed (also see Introduction). These modulations further imply that larval age and the composition of food may need to be taken into consideration in cross-laboratory comparisons of amino acid preference. Indeed, it has been reported that the concentration of salt in the food impacts larval salt preferences (Russell et al., 2011). In this context it seems significant that in all three of our preference experiments, as well as in Croset et al. (2016), a robust preference was found only for aspartic acid. It would therefore seem wise to focus on aspartic acid for future studies of single amino acid processing in the larva.

It remains to be determined whether avoidance of amino acids is biologically relevant. On the one hand, the observation of avoidance of different amino acids in different experiments may call for caution. On the other hand, as we measure animals' behaviour towards only one particular concentration of amino acids, it is possible that larvae may express avoidance of additional amino acids at different concentrations; likewise, the amino acids we found to be avoided may be preferred at another concentration. Also, Ignell et al. (2010) found that when individual amino acids mixed with sugar were tested for preference in the mosquito, several amino acids reduced preference. This effect, as well as an inhibition of sucrose feeding by amino acids, was lately observed in larval *Drosophila* as well (Croset et al., 2016). Thus, negative-valence effects of amino acids may well be a biological reality.

In any event, given that amino acid preferences can be modulated by larval stage and starvation, it will be interesting to see whether larvae adjust their feeding strategies to their nutritional status as shown in adult flies, locusts and rats (Hawkins et al., 1994; Simpson and White, 1990; Simpson et al., 1991; Toshima and Tanimura, 2012; Toshima et al., 2014). Indeed, Bjordal et al. (2014) reported that larval *Drosophila* reject a diet lacking a particular subset of amino acids, some, but not all, of which had previously been classified as essential (Sang and King, 1961).

It is striking that although amino acids differ in their innate attractiveness (Fig. 1) their strength as a reward does not (Fig. 2). Similar differences between innate behaviour and mnemonic effect have also been reported for other taste reinforcers, of both a rewarding and a punishing kind (Niewalda et al., 2008; Schipanski et al., 2008; El-Keredy et al., 2012). Maybe the most revealing case is quinine: innate avoidance and the punishing effect of quinine are mediated by distinct sets of gustatory sensory neurons (Apostolopoulou et al., 2014a). Thus, our results prompt the question of whether innate preference and the rewarding effects of amino acids likewise rely on different sensory input channels.

Recently we have shown that larvae form odour-tastant associative memories that are specific between fructose and aspartic acid (Schleyer et al., 2015). The discovery of 19 further amino acid rewards now calls for tests of specificity among different amino acids.

Together, the present analyses of preferences for, and learning of, amino acids will facilitate the ongoing search for the receptor molecules, gustatory sensory neurons, and internal, homeostatic amino acid sensors in larval *Drosophila*.

## MATERIALS AND METHODS

### Preference experiments

#### Animals

The flies were maintained on Kyushu standard food medium (Water 1 l, corn meal 50 g, glucose 100 g, dry yeast 40 g, wheat germ powder 32 g, agar 7.7 g, propionic acid 5 ml, methyl paraben 1.17 g) at 25°C, and under a 12-h light/dark cycle. To obtain first-instar larvae, adult flies were allowed to lay eggs on apple juice-soaked filter papers on which nylon mesh was placed for 4 h from the start of the dark period; larvae were collected at 23 h from the start of this 4-h egg laying time window. Third-instar larvae were collected from food vials 5 days after egg-laying. Animals were rinsed with distilled water before tests.

#### Chemicals

Agar (purified powder) was obtained from Sigma-Aldrich (St Louis, MO, USA). As tastants, D-fructose (CAS: 57-48-7, Wako Pure Chemical Industries, Ltd., Osaka, Japan), as well as L-alanine (ALA; CAS: 56-41-7), L-arginine monohydrochloride (ARG; CAS: 1119-34-2), L-asparagine monohydrate (ASN; CAS: 5794-13-8), L-aspartic acid (ASP; CAS: 56-84-8), L-cysteine monohydrochloride hydrate (CYS; CAS: 7048-04-6), L-histidine monohydrochloride (HIS; CAS: 645-35-2), L-isoleucine (ILE; CAS: 73-32-5), L-leucine (LEU; CAS: 61-90-5), L-lysine monohydrochloride (LYS; CAS: 657-27-2), L-glutamine (GLN; CAS: 56-85-9), L-glutamic acid (GLU; CAS: 56-86-0), L-glycine (GLY; CAS: 56-40-6), L-methionine (MET; CAS: 63-68-3), L-phenylalanine (PHE; CAS: 63-91-2), L-proline (PRO; CAS: 147-85-3), L-serine (SER; CAS: 56-45-1), L-threonine (THR; CAS: 72-19-5), L-tryptophan (TRP; CAS: 73-22-3), L-tyrosine (TYR; CAS: 60-18-4) and L-valine (VAL; CAS: 72-18-4) were used at concentrations of 10 mmol l^−1^ (TYR was used in 0.5 mmol l^−1^ due to low solubility). Amino acids were obtained from Wako Pure Chemical Industries, Ltd., Osaka, Japan (TYR, SER, CYS, VAL), Sigma-Aldrich, St Louis, MO, USA (ILE, PRO) or Nacalai Tesque, Kyoto, Japan (ALA, ARG, ASN, ASP, LEU, LYS, GLN, GLY, MET, PHE, THR, TRP, TYR).

#### Behaviour

All Petri dishes were prepared one day before behavioural experiments (35 mm diameter for first-instar and 90 mm for third-instar larvae). They were prepared consisting of an inner circle (10 mm diameter for first-instar larvae and 27.6 mm diameter for third-instar larvae) with 1% agar plus the respectively indicated tastant, and an outer circle with only the 1% agar. We collected 10-15 larvae, placed them onto the inner circle and after 30 min determined the number of larvae (#) in the inner circle and the total number of larvae on the Petri dish, as well as the ratio of these numbers. As a negative control, we prepared dishes with plain agar also in the inner circle. We calculated tastant preference as:
(1)
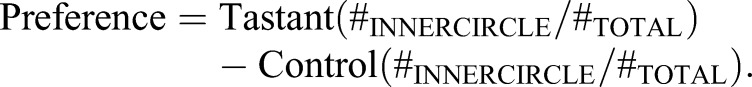


Thus, positive values indicate that more animals were found in a tastant-containing inner circle than in a pure agar-containing inner circle (Control). In other words, positive values indicate preference and negative values indicate avoidance of the tastant. This assay turned out to be more sensitive in detecting concentration-dependent levels of fructose preference than the split Petri dish assay used, for example, by Miyakawa (1982) and Schipanski et al. (2008) (Fig. S1). The design of the Petri dishes allows for some diffusion of the tastant into the outer ring, thus establishing a taste gradient at the border. Such a gradient may be used by the animals to orient on the Petri dish and to stay within their preferred area. Thus, although our assay measures the animals' distribution in a binary way (inner circle versus outer circle), it may well reflect a gradual distribution of the animals along a gradient rather than a binary choice.

#### Statistical analyses

The data were compared across multiple groups by Kruskal–Wallis tests; in case of significance, data from individual groups were compared to zero by one-sample sign-tests and corrected for multiple testing according to Bonferroni–Holm. For pairwise comparisons between groups, Mann–Whitney U-tests were performed. Bar plots and error bars represent means±s.e.m.

### Learning experiments

#### Animals

The flies were maintained on Magdeburg standard food medium (water 1 l,polenta 173.5 g, malt 86.7 g, molasses 54.2 g, soy flour 12.0 g, yeast 22.3 g, agar 9.0 g, and methyl paraben 3.0 g) at 25°C, and under a 12-h light/dark cycle. Third-instar larvae were collected from food vials 5 days after egg-laying. Animals were rinsed with distilled water before tests.

#### Chemicals

The same set of 20 amino acids was used as described above (some with deviating CAS numbers: ARG: 74-79-3; ASN: 70-47-3; CYS: 52-90-4; HIS: 71-00-1), obtained from Sigma-Aldrich (Seelze, Germany). The amino acids were added to 1% agarose (Roth, Karlsruhe, Germany) and poured into Petri dishes of 90 mm diameter. As odours, we used *n*-amyl acetate (AM; CAS: 628-63-7; Merck, Darmstadt, Germany), diluted 1:50 in paraffin oil, and undiluted 1-octanol (OCT; CAS: 111-87-5; Sigma-Aldrich); these were filled into custom-made Teflon containers that allowed evaporation of the odour.

#### Behaviour

Experiments followed standard procedures (Gerber et al., 2013). Thirty larvae were trained by three cycles of paired presentation of, for example, AM with the respectively indicated amino acid and OCT with a tasteless agarose substrate (AM+/OCT; in half of the cases the sequence was reversed: OCT/AM+). The larvae were then transferred to a tasteless test dish and given the choice between the two odours. After 3 min, the number of larvae (#) on either side was determined, and preference was calculated as:
(2)
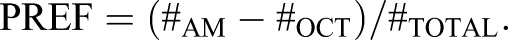


Thus, positive preference values indicate that the animals preferred AM.

For each group of larvae trained AM+/OCT (or OCT/AM+), a second group was trained reciprocally, i.e. AM/OCT+ (or OCT+/AM, respectively). From two reciprocally trained groups of animals we calculated an associative performance index as:
(3)
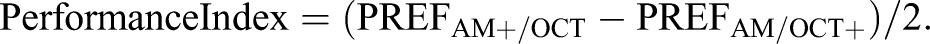


Positive values thus indicate appetitive and negative values indicate aversive associative memory.

#### Statistical analyses

The data were compared across multiple groups by Kruskal–Wallis tests; in case of significance, data from individual groups were compared to zero by one-sample sign-tests, corrected for multiple testing according to Bonferroni–Holm. For pairwise comparisons between groups, Mann–Whiney U-tests were performed. Box plots show the median as the middle line, and 25% and 75%, and 10% and 90% quantiles as box boundaries and whiskers, respectively.

## References

[BIO020412C1] Apostolopoulou A. A., Mazija L., Wüst A. and Thum A. S. (2014a). The neuronal and molecular basis of quinine-dependent bitter taste signaling in *Drosophila* larvae. *Front. Behav. Neurosci.* 8, 6 10.3389/fnbeh.2014.00006 24478653 PMC3902218

[BIO020412C2] Apostolopoulou A. A., Hersperger F., Mazija L., Widmann A., Wüst A. and Thum A. S. (2014b). Composition of agarose substrate affectes behavioral output of *Drosophila* larvae. *Front. Behav. Neurosci.* 8, 11 10.3389/fnbeh.2014.00011 24478658 PMC3904111

[BIO020412C3] Apostolopoulou A. A., Rist A. and Thum, A. S (2015). Taste processing in *Drosophila* larvae. *Front. Integr. Neurosci.* 9, 50 10.3389/fnint.2015.00050 26528147 PMC4602287

[BIO020412C4] Bjordal M., Arquier N., Kniazeff J., Pin J. P. and Léopold P. (2014). Sensing of amino acids in a dopaminergic circuitry promotes rejection of an incomplete diet in *Drosophila*. *Cell* 156, 510-521. 10.1016/j.cell.2013.12.024 24485457

[BIO020412C5] Croset V., Schleyer M., Arguello J. R., Gerber B. and Benton R. (2016). A molecular and neuronal basis for amino acid sensing in the *Drosophila* larva. *Sci. Rep.* 6, 34871 10.1038/srep34871 27982028 PMC5159833

[BIO020412C6] El-Keredy A., Schleyer M., König C., Ekim A. and Gerber B. (2012). Behavioural analyses of quinine processing in choice, feeding and learning of larval *Drosophila*. *PLoS ONE* 7, e40525 10.1371/journal.pone.0040525 22802964 PMC3393658

[BIO020412C8] Gerber B. and Stocker R. F. (2007). The *Drosophila* larva as a model for studying chemosensation and chemosensory learning: a review. *Chem. Senses.* 33, 563-573. 10.1093/chemse/bjl030 17071942

[BIO020412C9] Gerber B., Stocker R., Tanimura T. and Thum A. S. (2009). Smelling, tasting, learning: *Drosophila* as a study case. *Results. Probl. Cell. Differ.* 47, 139-185. 10.1007/400_2008_9 19145411

[BIO020412C10] Gerber B., Biernacki R. and Thum J. (2013). Odor-taste learning assays in *Drosophila* larvae. *Cold Spring Harb Protoc.* 2013, 213-223. 10.1101/pdb.prot071639 23457337

[BIO020412C11] Hawkins R. L., Inoue M., Mori M. and Torii K. (1994). Lysine deficient diet and lysine replacement affect food directed operant behavior. *Physiol. Behav.* 56, 1061-1068. 10.1016/0031-9384(94)90344-1 7824572

[BIO020412C12] Ignell R., Okawa S., Englund J.-E. and Hill S. R. (2010). Assessment of diet choice by the yellow fever mosquito *Aedes aegypti*. *Physiol. Entomol.* 35, 274-286. 10.1111/j.1365-3032.2010.00740.x

[BIO020412C13] Kim Y. S. and Smith B. H. (2000). Effect of an amino acid on feeding preferences and learning behavior in the honey bee, *Apis mellifera*. *J. Insect Physiol.* 46, 793-801. 10.1016/s0022-1910(99)00168-7 10742528

[BIO020412C14] Kim H., Choi M. S., Kang K. J. and Kwon J. Y. (2015). Behavioral analysis of bitter taste perception in *Drosophila* larvae. *Chem. Senses.* 41, 85-94. 10.1093/chemse/bjv061 26512069

[BIO020412C15] Miyakawa Y. (1982). Behavioural evidence for the existence of sugar, salt and amino acid taste receptor cells and some of their properties in *Drosophila* larvae. *J. Insect. Physiol.* 28, 405-410. 10.1016/0022-1910(82)90066-X

[BIO020412C16] Niewalda T., Singhal N., Fiala A., Saumweber T., Wegener S. and Gerber B. (2008). Salt processing in larval *Drosophila*: choice, feeding, and learning shift from appetitive to aversive in a concentration-dependent way. *Chem. Senses.* 33, 685-692. 10.1093/chemse/bjn037 18640967 PMC2565773

[BIO020412C17] Russell C., Wessnitzer J., Young J. M., Armstrong J. D. and Webb B. (2011). Dietary salt levels affect salt preference and learning in larval *Drosophila*. *PLoS ONE.* 6, e20100 10.1371/journal.pone.0020100 21687789 PMC3105986

[BIO020412C18] Ryuda M., Tsuzuki S., Tanimura T., Tojo S. and Hayakawa Y. (2008). A gene involved in the food preferences of larval *Drosophila melanogaster*. *J. Insect. Physiol.* 54, 1440-1445. 10.1016/j.jinsphys.2008.08.006 18773904

[BIO020412C19] Sang J. H. and King R. C. (1961). Nutritional requirements of axenically cultured *Drosophila melanogaster* adults. *J. Exp. Biol.* 38, 793-809.

[BIO020412C20] Schipanski A., Yarali A., Niewalda T. and Gerber B. (2008). Behavioral analyses of sugar processing in choice, feeding, and learning in larval *Drosophila*. *Chem. Senses.* 33, 563-573. 10.1093/chemse/bjn024 18511478 PMC2467463

[BIO020412C21] Schleyer M., Miura D., Tanimura T. and Gerber B. (2015). Learning the specific quality of taste reinforcement in larval *Drosophila*. *Elife* 4, e04711 10.7554/eLife.04711 PMC4302267 25622533

[BIO020412C22] Simpson S. J. and White P. R. (1990). Associative learning and locust feeding: evidence for a ‘learned hunger’ for protein. *Anim. Behav.* 40, 506-513. 10.1016/S0003-3472(05)80531-7

[BIO020412C23] Simpson S. J., James S., Simmonds M. S. and Blaney W. M. (1991). Variation in chemosensitivity and the control of dietary selection behavior in the locust. *Appetite* 17, 141-154. 10.1016/0195-6663(91)90069-5 1763906

[BIO020412C24] Toshima N. and Tanimura T. (2012). Taste preference for amino acids is dependent on internal nutritional state in *Drosophila melanogaster*. *J. Exp. Biol.* 215, 2827-2832. 10.1242/jeb.069146 22837455

[BIO020412C25] Toshima N., Hara C., Scholz C.-J. and Tanimura T. (2014). Genetic variation in food choice behaviour of amino acid-deprived *Drosophila*. *J. Insect. Physiol.* 69, 89-94. 10.1016/j.jinsphys.2014.06.019 25010547

